# Extreme Sensitivity of Fitness to Environmental Conditions: Lessons from #1BigBatch

**DOI:** 10.1007/s00239-023-10114-3

**Published:** 2023-05-27

**Authors:** Grant Kinsler, Kara Schmidlin, Daphne Newell, Rachel Eder, Sam Apodaca, Grace Lam, Dmitri Petrov, Kerry Geiler-Samerotte

**Affiliations:** 1 Stanford University, Stanford, USA; 2 Center for Mechanisms of Evolution, Arizona State University, Tempe, USA; 3 School of Life Sciences, Arizona State University, Tempe, USA

**Keywords:** Fitness, Barcodes, Batch effects, Index misassignment, Reproducibility, Experimental evolution

## Abstract

The phrase “survival of the fittest” has become an iconic descriptor of how natural selection works. And yet, precisely measuring fitness, even for single-celled microbial populations growing in controlled laboratory conditions, remains a challenge. While numerous methods exist to perform these measurements, including recently developed methods utilizing DNA barcodes, all methods are limited in their precision to differentiate strains with small fitness differences. In this study, we rule out some major sources of imprecision, but still find that fitness measurements vary substantially from replicate to replicate. Our data suggest that very subtle and difficult to avoid environmental differences between replicates create systematic variation across fitness measurements. We conclude by discussing how fitness measurements should be interpreted given their extreme environment dependence. This work was inspired by the scientific community who followed us and gave us tips as we live tweeted a high-replicate fitness measurement experiment at #1BigBatch.

**Supplementary Information:**

The online version contains supplementary material available at 10.1007/s00239-023-10114-3.

## Introduction

Measuring the relative fitness of mutant microbial strains has revolutionized our understanding of functional genomics and basic cell biology. For example, comparing the fitness of microbial strains with different gene deletions taught us about the function of thousands of genes (Giaever et al. 2002; Breslow et al. 2008; Gresham et al. 2011), which genes work together (Costanzo et al. 2016), and about the genetic programs that allow cells to respond to challenges like drugs, high temperature, or nutrient deprivation (Gasch et al. 2000; Slavov and Botstein 2011; She and Jarosz 2018). Precisely quantifying the fitness of microbial populations is also of interest to evolutionary biologists, for example, those surveying the fitness effects of adaptive mutations (Levy et al. 2015; Venkataram et al. 2016; Kinsler et al. 2020), deleterious mutations (Wloch et al. 2001; Geiler-Samerotte et al. 2011; Johnson et al. 2019), or combinations of mutations (Flynn et al. 2020; Aggeli et al. 2021; Bakerlee et al. 2022). Further, measuring fitness of infectious microbial populations is of interest in evolutionary medicine (Brown et al. 2010; Nichol et al. 2019; Dunai et al. 2019) and measuring the fitness of engineered microbes is critical in industries focused on bioproduct production (Chubukov et al. 2016).

Given the importance of measuring microbial fitness in diverse fields, it is frustrating that we cannot measure fitness more precisely. Reported precision remains capped at detecting fitness differences on the order of 0.1–1% (Gallet et al. 2012; Leon et al. 2018; Duveau et al. 2018). Achieving this level of precision requires averaging results across many replicates. If replicates suffer from common reproducibility issues (Lithgow et al. 2017; Kinsler et al. 2020; Worthan et al. 2023), the resulting estimates of precision may be inflated (Box 1). On the other hand, natural selection can distinguish fitness differences that are orders of magnitude smaller than we can measure in the laboratory, depending on effective population size (Ohta 1973; Lynch and Conery 2003). This creates a problem for scientists: how do we understand the fitness effects of mutations if many of these effects are too small for us to measure precisely?

### Methods to Measure Fitness and Limitations on Their Precision

There are many reasons why the methods we use to measure fitness are limited in their precision. One common way to compare fitness across microbial strains is to measure each strain’s growth rate, in other words, the rate at which cells divide to make more cells. The change in cell density over time is often measured by the increase in optical density of liquid culture (Ram et al. 2019) or the increase in colony size on an agarose plate (She and Jarosz 2018) or a glass bottom plate (Levy et al. 2012; Sartori et al. 2021). This method often has limitations on precision, one being that measuring cell density is not as precise as counting individual cells and another being that each microbial strain is usually grown separately and is separately affected by any biological or technical noise. A less common method to measure microbial growth rate is to track the level of a protein or transcript that controls growth, rather than measuring growth itself (Brauer et al. 2008; Geiler-Samerotte et al. 2013; Scott et al. 2014; Wu et al. 2022), although this method suffers the same limitations as those above.

Many consider the gold standard for measuring fitness to be an experiment where strains are competed in the same vessel (Hegreness et al. 2006; Kao and Sherlock 2008; Geiler-Samerotte et al. 2011; Ram et al. 2019). A benefit of this method over others is that all strains are grown simultaneously in the same well-mixed media in the same vessel and thus subject to the same exact environment. Further, fitness is defined more broadly than population growth rate because it includes differential survival in conditions where growth is not proceeding exponentially or is halted (Ram et al. 2019). Finally, another benefit of competition experiments is that, in many implementations of this method, individual cells are counted. This is more precise than tracking changes in cellular density over time using crude parameters, like optical density, as long as enough cells are sampled.

Despite their advantages, fitness competitions still suffer from noise. For example, some competitions label competing strains with fluorescent proteins or genetic markers distinguishable in the presence of a drug or carbon source. Then, the fraction of cells labeled with each marker are counted as they change over time (Hegreness et al. 2006; Breslow et al. 2008; Kao and Sherlock 2008; Geiler-Samerotte et al. 2011; Gallet et al. 2012; Lenski 2017). These methods report percent error ranging from 0.01 to 1%. Sources of noise include sampling noise, which worsens when fewer cells from each fraction are counted, and cell assignment noise, which worsens, for example, when fluorescent reporters are chosen that are more difficult to distinguish from each other (Gallet et al. 2012). More recent methods utilize “**DNA Barcodes**” to distinguish strains (Levy et al. 2015; Venkataram et al. 2016; Kinsler et al. 2020; Bakerlee et al. 2021). These are short regions of the genome that are unique to a given strain and flanked by consensus sequences such that all barcodes can be amplified using the same primer pair. Next-generation sequencing allows for tracking the frequency of each DNA barcode over time with incredibly high throughput such that millions of individual cell barcodes can be counted at each timepoint. This high throughput can reduce sampling noise.

Here, we examine fitness estimates that were generated in previous studies by quantifying how the DNA barcode that labels a particular strain changes in frequency over time relative to a reference (Venkataram et al. 2016; Li et al. 2018b; Kinsler et al. 2020). This may yield more precise fitness estimates than deep mutational scanning methods that measure the fold change in a barcode’s frequency, rather than inferring fitness by modeling a barcode’s rate of change over many time points (Li et al. 2018a). As in fitness competitions utilizing fluorescent markers, inadequate sampling can increase noise. In the data we present here, this type of sampling noise has been minimized by sequencing barcodes at very high coverage.

### Even with High Sequence Coverage, We Observe “Noisy” Fitness Data

**Fig. 1:**
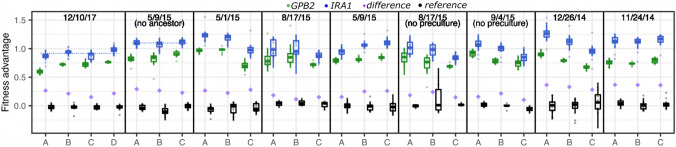
Each panel represents 3 to 4 replicate fitness competition experiments (labeled A, B, C, or D on the horizontal axis) that were performed in the same “batch” (i.e., on the same date following the same protocol). Any known batch-specific protocol modifications are listed in parentheses, although it is likely that minor differences exist between every batch due to the nature of wet lab work. Each point represents the fitness of a uniquely barcoded yeast lineage with a mutation in the *IRA1* (blue) or *GPB2* (green) gene or a reference strain possessing no mutations (black). Boxplots summarize the distribution across all lineages possessing mutations to the same gene, displaying the median (center line), interquartile range (IQR) (upper and lower hinges), and highest value within 1.5 × IQR (whiskers). Purple diamonds represent the average fitness difference, per replicate, between all lineages possessing *IRA1* vs. *GPB2* mutations. Dotted blue lines in the first two panels guide the eye to observe what we define as a “batch effect,” whereby the fitness of lineages possessing nonsense mutations in the *IRA1* gene differs across experiments performed on different days (Color figure online)

In this study, we investigate some sources of noise that contribute to variation in fitness measurements of barcoded strains. We focus on barcodes that received extremely high sequencing coverage to minimize sampling noise arising at the sequencing step. This allows us to dissect other potential sources of noise in barcoded fitness competitions in *S. cerevisiae*. We start by studying two sources of technical noise: (1) noise introduced while barcodes are prepared for sequencing and (2) index misassignment during barcode sequencing (Sinha et al. 2017). Despite common intuition (Levy et al. 2015; Venkataram et al. 2016; KerryGeilerSamerotte 2018a), we find that only the latter is a major source of noise. We suggest an index scheme to help reduce this type of error. Next, we turn our focus to distinguish noise that affects barcode frequencies within a single competition experiment from noise across replicate experiments. We find higher variation in fitness across replicate experiments, particularly those performed on different days. For example, certain adaptive mutants have fitnesses that range from 180% of the reference in one replicate to 220% in others (Fig. 1). This type of variation is often called “**batch effects**,” i.e., systematic differences that arise between very similar experiments performed at different times. These batch effects may reflect biologically relevant but subtle changes in environmental conditions that influence fitness, for example, slight differences in temperature, media composition, humidity, shape of the culture vessel, and shaking speed.

This observation that fitness may be extremely sensitive to environmental context makes us question how to obtain and interpret fitness measurements. Should we focus on minimizing replicate and batch effects, perhaps using incubators with extremely low variation in temperature or ultra-precise scales for media preparation? Or should we embrace variation in fitness and search for new insights about genotype-by-environment interactions by studying the way fitness varies across replicates and batches (Kinsler et al. 2020)? We argue that both approaches have value. We hope the findings of our open-science endeavor to generate more precise fitness measurements (KerryGeilerSamerotte 2017; Kinsler 2017) will act as a user’s guide for others quantifying fitness with DNA barcodes. We also hope it will inspire important questions about how to measure and report fitness given its sensitivity to subtle environmental change and will fuel the larger discussion on how to conduct reproducible and precise laboratory studies.

## Results

### Choosing Focal Yeast Strains to Study Noise in Barcoded Fitness Competitions

Our goal is to dissect the sources of noise that contribute to variation in fitness beyond sources that are easy to understand such as the sampling noise that is introduced when barcodes are sequenced at low coverage. Thus, when possible, we focus on variation in fitness for barcoded mutants that (1) are strongly adaptive and (2) have been sequenced at extremely high coverage. We focus on strongly adaptive mutants because these increase in frequency over the course of a competition experiment, thus sequencing coverage continues to improve over time. Previous experimental evolutions utilizing DNA barcodes discovered two types of strongly adaptive mutants that commonly arise in baker’s yeast in response to glucose limitation: nonsense mutations to the *IRA1* gene and missense mutations to *GPB2* (Levy et al. 2015; Venkataram et al. 2016). We previously measured the fitness of these adaptive mutants, when pooled with at least 400 others, in 28 replicate experiments (Kinsler et al. 2020). In these studies, barcodes representing lineages possessing adaptive mutations in either *IRA1* or *GPB2* are sequenced an average of 37,690 or 13,321 times per timepoint, respectively. For comparison, previous barcoded fitness competitions aim for coverage thresholds that are smaller by orders of magnitude (100 to 200 reads per mutant per timepoint) (Venkataram et al. 2016; Kinsler et al. 2020). Despite very high sequencing coverage, we see a lot of variation in the fitness estimates for these mutants (Fig. 1). This inspired us to think about the major sources of noise that affect fitness estimation in barcoded competition experiments like the ones we performed previously (Kinsler et al. 2020).

### Motivation and Design for Studying Noise Introduced by DNA Extraction or Amplification

One source of noise that seemed like a potentially large contributor to variation in fitness is introduced when subsets of cells and molecules are sampled during preparation of barcodes for sequencing. In order to infer fitness from changes in barcode frequency over time, one must first extract DNA from a large number of cells at many timepoints (KerryGeilerSamerotte 2018b). Next, one must amplify the barcode region with primers that attach sequencing indices. Both of these steps can introduce imprecision into a fitness measurement. For example, if DNA is extracted from too few cells, or extracted in a biased way such that cells with certain mutations contribute an unfair quantity of DNA, fitness estimates may be imprecise or skewed. Similarly, if PCR is inefficient such that only a small number of barcodes are amplified in the early cycles, these barcodes will become overrepresented in a way that does not reflect their true abundance (i.e., PCR jackpotting). We utilize special DNA extraction and PCR protocols that are designed to avoid these issues (Levy et al. 2015; Venkataram et al. 2016; Kinsler et al. 2020). Still, previous work (Levy et al. 2015; Venkataram et al. 2016), as well as a poll of 100 scientists (KerryGeilerSamerotte 2018a), suggested that stochastic events during barcode extraction and amplification may be large contributors to noise in barcoded fitness competitions. On a personal note, when performing these tricky steps of sample preparation in the lab, we found ourselves constantly considering what, if any, noise or bias they were introducing into our data. Therefore, we were strongly motivated to investigate this potential source of noise.

**Fig. 2:**
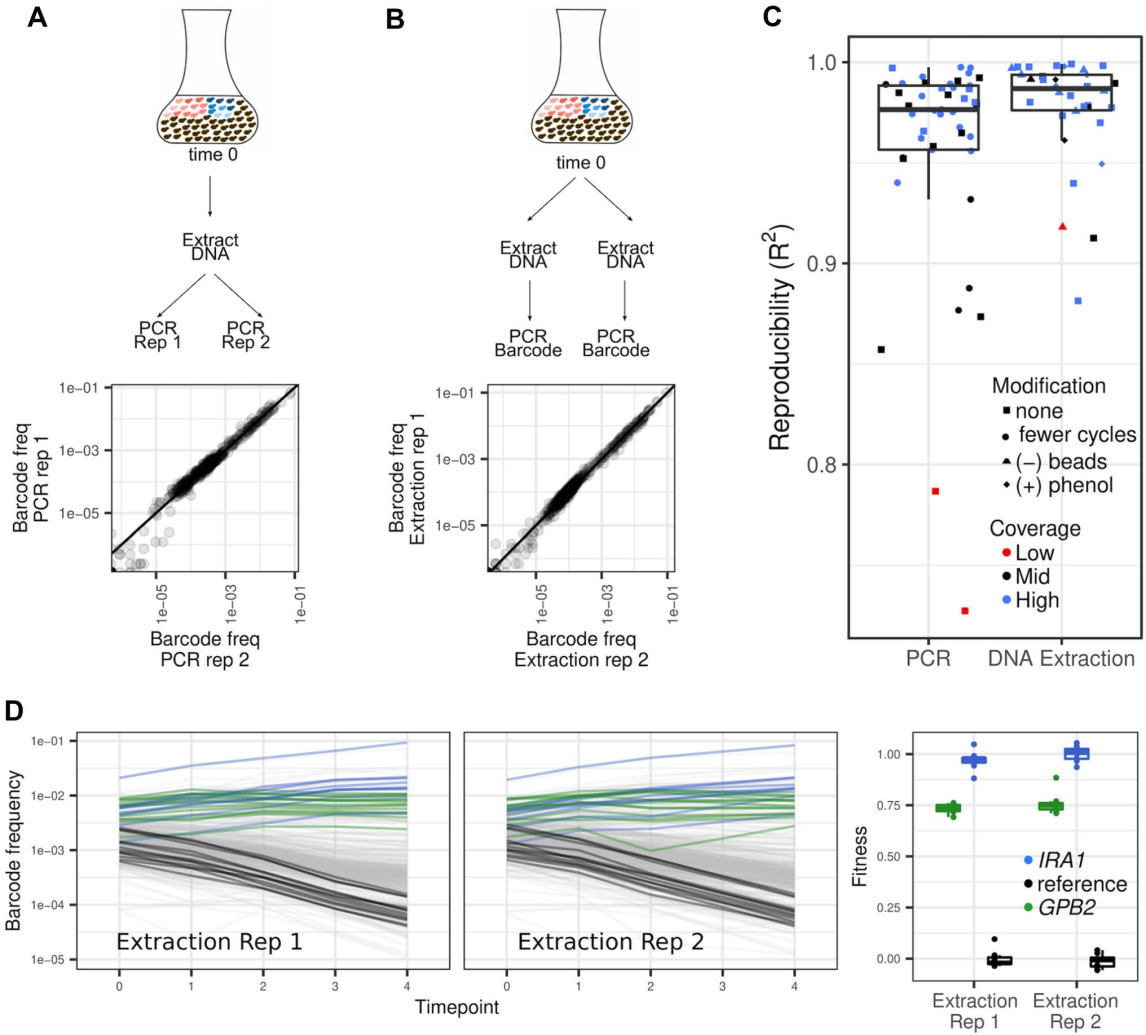
Stochastic noise generated during barcode extraction and amplification appear to contribute little noise to fitness estimation. **A** Schematic showing how we divided a sample to perform a PCR replicate and comparison of barcode frequencies for a pair of replicates where every point represents one of ~ 500 barcodes. **B** Schematic showing how we divided a sample to perform an extraction replicate and comparison of barcode frequencies for a pair of replicates where every point represents one of ~ 500 barcodes. **C** Reproducibility (R^2^) is greater than 0.9 for most pairs of PCR and extraction replicates. Modifications to the PCR or extraction procedure did not seem to affect reproducibility. Low sequencing coverage did often result in lower reproducibility. Boxplots summarize the distribution across all PCR or extraction replicates, displaying the median (center line), interquartile range (IQR) (upper and lower hinges), and highest value within 1.5 × IQR (whiskers). **D** Barcode frequency trajectories where each line represents the frequency of 1 of ~ 500 barcoded mutants through time. The trajectories labeled “Extraction Rep 1” and “Extraction Rep 2” look similar despite being composed of different technical replicates. When fitness is inferred from the log-linear slope of these trajectories, the inferences are also similar for strains possessing mutations in either *IRA1* or *GPB2*. Boxplots summarize the distribution across mutant lineages of the same type, displaying the median (center line), interquartile range (IQR) (upper and lower hinges), and highest value within 1.5 × IQR (whiskers)

To understand the contribution of these two sources of imprecision, we performed dozens of technical replicate experiments (KerryGeilerSamerotte 2018c; Kinsler 2018a) where we split cells from a single timepoint and either performed multiple replicate PCRs (Fig. 2A) or multiple replicate DNA extractions (Fig. 2B). Then, we compared the barcode frequencies from pairs of technical replicates to see how much noise (measured via R^2^) is introduced by PCR and by DNA extraction.

An important complicating factor is that we measure fitness as the log-linear rate of change in a barcode’s frequency over 5 time points (Venkataram et al. 2016; Li et al. 2018b, a; Kinsler et al. 2020). Previous work suggests this results in more accurate fitness estimates than does measuring the fold change in a barcode’s frequency across two time points (Li et al. 2018a). Unfortunately, we cannot estimate noise contributing to fitness measurements using the same well-established models as those that have been previously used to study fold change (Robinson et al. 2014). Our pairs of technical replicates only let us measure reproducibility at single time points. Understanding variation in fitness requires the additional step of understanding how noise introduced across all timepoints aggregates.

### DNA Extraction and Amplification do not Introduce Enough Noise to Explain Observed Fitness Variation

We found that stochasticity during PCR amplification usually contributes only a small amount of noise (Fig. 2C). In total, we compared 45 pairs of technical replicate PCRs. In some of these, both replicates included 27 PCR cycles; in others one replicate included fewer total cycles (22 v. 27). These deviations in cycle number did not appear to lead to greater imprecision (Fig. 2C) or bias (Fig. 2A). The typically high reproducibility we saw between PCR technical replicates could indicate that the two-step PCR protocol often used in barcoded fitness competitions is robust to effects like PCR jackpotting (Levy et al. 2015; Venkataram et al. 2016; Li et al. 2018b; Kinsler et al. 2020).

We found that stochasticity during DNA extraction also contributes only a small amount of noise (Fig. 2C). In total, we compared 34 pairs of technical replicate DNA extractions. In some of these, both replicates were performed following the exact same protocol. In others, one replicate was performed following a protocol that involved either a harsher treatment (i.e., adding phenol to help cells release their DNA) or a milder treatment (i.e., omitting glass beads that are used to break the cells). These modifications did not appear to affect reproducibility (Fig. 2C) or introduce bias (Fig. 2B). The high reproducibility we saw between technical replicates could indicate that our protocol is effective at extracting DNA from a very large number of yeast cells in an unbiased way (Kinsler et al. 2020).

Because DNA extraction is necessarily followed by PCR, any noise detected in our DNA extraction replicates also includes noise contributed during the PCR step (Fig. 2B). Therefore, we expected DNA extraction replicates to be either as noisy or noisier than PCR replicates. However, we observed the opposite. The reason is as follows. For a few of our technical replicates, we observed lower reproducibility (R^2^ < 0.9 for 6/45 pairs of PCR replicates). This lower reproducibility seems more common in cases where one replicate of the pair happened to receive low (on average < 20 reads per barcode) or moderate (on average 20–600 reads per barcode) sequencing coverage (Fig. 2C). These cases of lower coverage artificially reduce the average reproducibility among technical replicate PCRs relative to technical replicate DNA extractions.

While our data are suggestive that the DNA extraction and PCR steps do not introduce enough noise to explain the observed variation in fitness (Fig. 1), they do not allow us to directly estimate how much noise these sources contribute. Next, we wanted to roughly estimate how the aggregate noise introduced across many timepoints by these procedures would affect fitness estimates. To do so we studied frequency trajectories from the experiment where we performed DNA and PCR technical replicates on the largest number of timepoints (4 out of 5 timepoints) (Fig. 2D). We found similar fitness measurements were inferred from the barcode frequency trajectories comprising each set of technical replicates. This suggests that, when following the protocols we use here, developed in earlier work (Levy et al. 2015; Venkataram et al. 2016; Kinsler et al. 2020), noise from PCR amplification and DNA extraction has a minor effect on fitness precision (Fig. 2D).

### Motivation and Design for Studying Noise Introduced by Index Misassignment

**Fig. 3:**
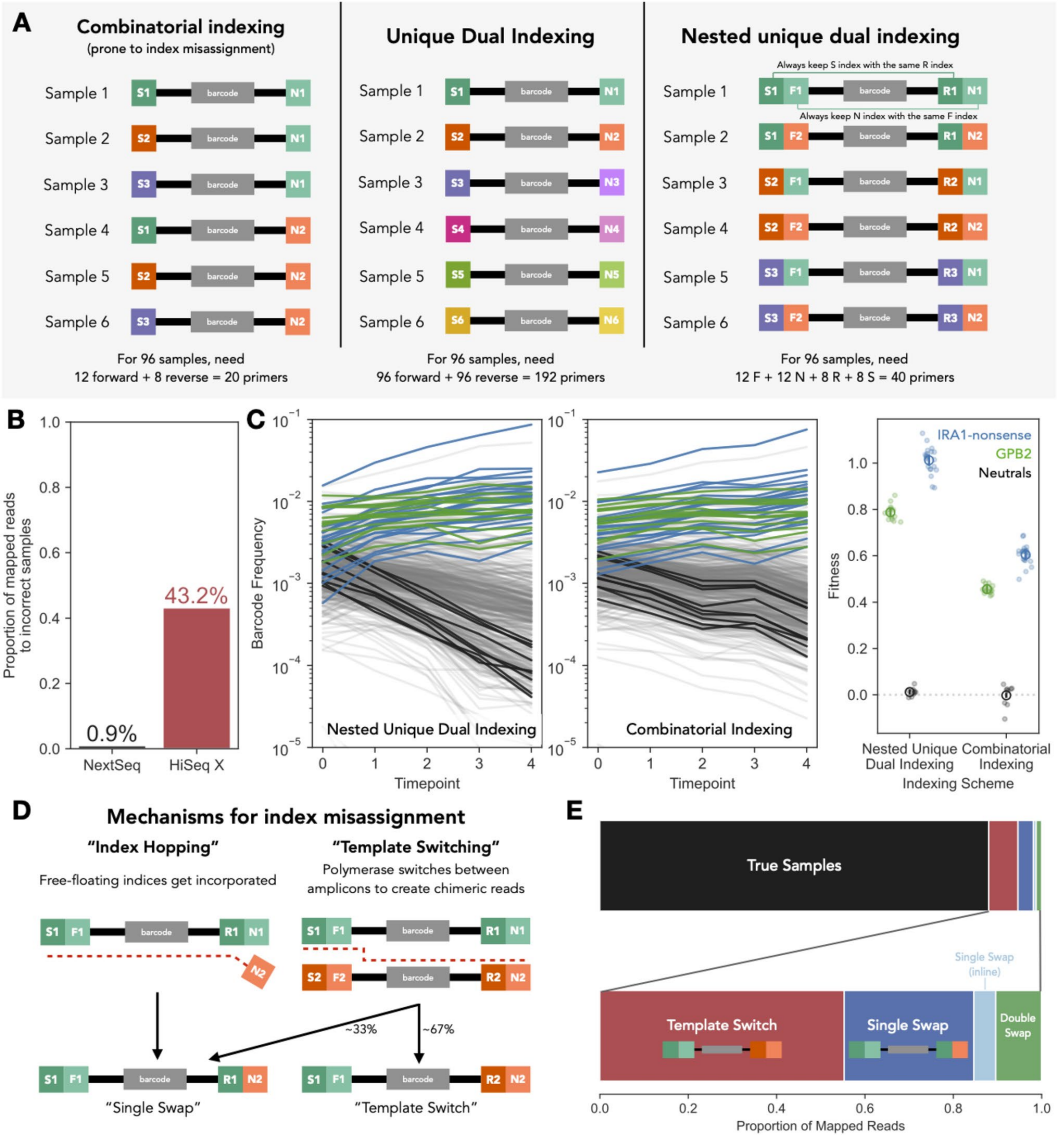
Template switching is a major source of incorrect sample assignment for barcode sequencing. **A** This panel shows three schemes for multiplexing samples onto the same sequencing lane. The first, “combinatorial indexing” assigns samples to many combinations of primers, allowing for many samples to be labeled with a relatively small number of indices. This scheme allows for index misassignment to incorrectly assign samples. There are several alternatives that reduce the effect of index misassignment and allow researchers to remove incorrect swaps from data. The most common scheme is “unique dual indexing,” where each sample uses a unique combination of forward and reverse indices. In this case, 192 primers are needed to label 96 samples. Another possible scheme, used in this paper, is nested unique dual indexing, where inline and Illumina indices are combined such that each inline index corresponds with a unique Illumina index on the other side of the amplicon molecule. This allows you to achieve unique dual indexing of 96 samples with 40 total primers. **B** Rates of incorrect sample index assignment are much higher on a HiSeq X (patterned flow cell) sequencing machine than NextSeq (non-patterned flow cell) for this set of 95 samples. **C** Undetected index misassignment can alter frequency trajectories and result in incorrect fitness inference. When index misassignment is detected using unique dual indexing (left panel), frequency trajectories show a more dramatic change in frequency, especially for neutral lineages (black lines). In contrast, if combinatorial indexing were to be used, index misassignment would fail to be detected, resulting in diminished changes in barcode frequency over time (middle panel). The right panel shows the fitness values inferred under each scenario. The colored dots denote the inferred fitness for each barcoded mutant. The open circle shows the mean across all mutants for a particular genotype, with the error bar denoting standard error. **D** There are two primary mechanisms for index misassignment. One mechanism (depicted on left) is “index hopping” due to misincorporation of free-floating index primers present in the library. This results in reads that differ by a single index from correct index combinations. Another mechanism (depicted on right) is “template switching” due to switching of the template molecule during the amplification step of sequencing. This results in chimeric reads, where each end of the molecule matches a correct index combination but do not belong together. If the template switching event occurs in the middle of the amplicon (~ 67% of the time if the events are uniformly distributed), then we will observe combinations of otherwise correct pairs. If the template switching event occurs between the Illumina and inline indices (~ 33% of the time), then we will identify a “single swap” readout indistinguishable from the product generated via “index hopping.” **E** For a library of 8 samples utilizing a nested unique dual-indexing scheme (panel A) consisting of 32 individual indices (such that each index was only used once), we can analyze which mechanisms caused index misassignment by looking at whether incorrectly indexed reads have one or more misplaced indices. The top stacked bar plot shows the proportion of reads where there was no index misassignment (black) and the proportion where indices were misassigned (colored). The bottom stacked bar plot shows the proportion of reads with misassigned indices that belong to each category of index misassignment. We find that template switching (red) is the most commonly observed mechanism of index misassignment, followed by single-index swaps of Illumina indices (dark blue) and double swaps (green). Single-index swaps of inline indices (light blue) occur at extremely low rates (Color figure online)

Another source of noise we investigated comes from index misassignment during next-generation sequencing. In order to combine several samples on a single lane of sequencing, researchers often make use of index primers that label each sample on the lane. In the past, combinatorial indexing schemes have been used to identify samples such that each pair of indices uniquely labels a sample (Fig. 3A). However, previous work, with relatively diverse RNASeq libraries, has shown platforms with patterned flow cells that utilize Illumina’s ExAmp chemistry (e.g., HiSeq 4000, HiSeqX, NovaSeq) have substantial rates of misassigned indices such that 5–10% of sequencing reads are misassigned (Sinha et al. 2017). This means that a substantial number of reads from one sample can appear to belong to another sample, causing errors with downstream analysis. These errors are especially problematic for analyses relying on quantitative measurements of abundances (e.g., barcode sequencing, RNASeq, or other amplicon-based sequencing, such as 16S, deep mutational scanning, and massively parallel reporter assays). Unfortunately, most previous studies of index misassignment have focused on RNASeq, rather than barcode sequencing.

Until recently, most of our barcoded fitness competitions were being sequenced on non-patterned flow cells (e.g., Nextseq) (Venkataram et al. 2016) that have not been reported to suffer from high rates of misassigned indices. However, patterned flow cells that utilize ExAmp chemistry allow for much greater throughput at lower cost per read. Thus, we were strongly motivated to switch technologies. We designed an experiment to understand the frequency of index misassignment in barcode sequencing, as well as the underlying mechanisms and potential solutions. All of the data reported in Fig. 1 were generated either using an unpatterned flow cell or after implementing our solution to the index misassignment problem. Although index misassignment is thus not a major contributor to the fitness variation reported in Fig. 1, we still report our findings about the rate of misassignment on patterned flow cells and our solution to the problem below.

In order to quantify the extent to which this misassignment affects amplicon libraries, we devised a nested dual-indexing approach that combines the use of inline indices and Illumina index primers. Specifically, we carry out two steps of PCR, the first of which attaches inline indices (denoted by F and R in Fig. 3A) to each side of the amplicon, followed by a second step which attaches Illumina index primers (denoted by N and S in Fig. 3A) which also contain the P5 and P7 sequences which bind to the flow cell. In this scheme, we ensure that each inline index is only paired with a specific Illumina index on the other side of the molecule (i.e., R1 is always paired with S1, F1 with N1, R2 with S2, etc.) (Fig. 3A). This allows us to detect index misassignment events when one or more of the inline indices do not match their paired Illumina index.

### Template Switching on Patterned Flow Cells Appears to be a Major Source of Noise

First, we ran a single pooled library of 95 samples (consisting of all allowed combinations of primers except one) on one lane of NextSeq (unpatterned) and one of HiSeq X (patterned). After demultiplexing the reads and mapping the reads to the template sequence, we find that only 1% of mapped NextSeq reads were assigned to incorrect combinations of indices. In contrast, 43% of mapped HiSeq X reads were assigned to incorrect combinations of indices (Fig. 3B). This is much larger than the previously reported value of 5–10% of reads misassigned for diverse RNASeq or WGS libraries (Illumina 2017; Sinha et al. 2017).

These very high rates of index misassignment can result in substantial effects on fitness measurement experiments. For example, if reads from a late timepoint are misassigned as coming from an early time point, lineages with high fitness (and thus high frequency in later time points) can have underestimated fitness effects, because their early time point frequency will be overestimated (Fig. 3C). Additionally, high rates index misassignment can result in frequency trajectories that “zig-zag” instead of reflecting constant frequency change overtime, resulting in very noisy fitness estimates (KerryGeilerSamerotte 2018d; Kinsler 2018b).

Why is it that our barcode libraries have much higher rates of index misassignment than previous reports focusing on RNASeq? There are two main mechanisms by which index misassignment could occur. First, free-floating indices present in the library could become incorporated into molecules initially tagged with another index, resulting in “index-hopping” (Fig. 3D; left). This mechanism has been identified as the primary driver of index misassignment in diverse libraries by Illumina (2017), with the recommended solution being to ensure libraries are free of unincorporated primers prior to sequencing in addition to unique dual indexing. Our experimental design can distinguish this type of index misassignment because this process should primarily occur through swapping of the Illumina index primers (which contain the flow cell-binding sequences; S and N in Fig. 3A rightmost panel), rather than inline indices (F and R in Fig. 3A rightmost panel). Therefore, we can identify these events by observing reads where all of the indices match an included sample except a single S or N Illumina index—we will refer to these events as “single swaps” (Fig. 3D; left).

A second mechanism may be that index misassignment occurs via “template switching” events, where the polymerase jumps between two homologous sequences to create chimeric sequences where each end is from distinct original molecules (Fig. 3D; right). Given that the diverse barcodes sequenced in amplicon libraries often include a homologous region that is identical between all molecules (Levy et al. 2015; Wong et al. 2016; Adamson et al. 2016; Najm et al. 2017; Gordon et al. 2020), this mechanism of swapping could represent a more severe problem for barcoders. In the case of our experimental design to detect index misassignment, if the swap occurs in the 167-bp homologous region between the inline indices, we would expect to find mismatched index pairs. In other words, the index pair on one end of the molecule (i.e., S and F) would match one sample while index pairs on the other end of the molecule (R and N) would match a different sample (Fig. 3D; right). However, if the template switching event occurs in one of the two 40-bp regions between the inline and Illumina indices, we cannot distinguish whether these events are due to template switching or index hopping, as these will also appear in our data as “single swaps.”

To understand which of these possible mechanisms drives increased index misassignment in our study, we deeply sequenced a library of 8 samples that were uniquely indexed using our nested unique dual-index scheme (Fig. 3A) such that each individual index was used with only a single sample (Kinsler 2018c). By examining the frequencies of each type of unexpected index combination (Fig. 3D), we gain insight about the mechanism underlying index misassignment. However, an important note is that, while this experimental design can tease out the *mechanism* of index misassignment, it is underpowered to measure the *rate* of index misassignment. The reason is that when only a few expected combinations of indices are used (e.g., 8), many index swapping events will occur between two sequences with the same indices. These swapping events go undetected in our experiment. While these events do not result in index misassignment, swapping within samples that have identical indices can represent a major problem in other studies (Box 2).

Sequencing this pooled library on a HiSeq X machine, we found that 88% of our mapped reads had correct combinations of indices (Fig. 3E). Again, we suspect the reason this experiment found only 12% of reads were misassigned, as opposed to 43% in our previous experiment is that this experiment is underpowered to measure the *rate* of index misassignment. Of the 12% misassigned reads, 55% (6.6% of the total mapped reads) were likely products of template switching events (Fig. 3D; right), while 29% of the misassigned reads (3.5% of total mapped reads) were the product of single swaps of an Illumina index (Fig. 3D; left). These single swaps are either due to incorporation of free-floating indices or template switching in the constant region between the inline and Illumina indices. Interestingly, the ratio of observed single swaps to definitive template switching events (29%/55% = 52%) is close to the ratio you would expect if template switching was the only mechanism of index misassignment and the location of the template switching event is equally likely across the entire amplicon sequence (49%). This suggests that most of these single swaps may be due to template switching events rather than “index hopping” events driven by incorporation of errant primers. In addition to these two prominent swapping events, 1.2% of total reads were double swaps (likely reflecting either multiple independent swapping events resulting in incorrect index combinations or residual contamination of some kind) and 0.5% of the total mapped reads were single swaps of an inline index.

These data suggest that template switching (Fig. 3D; right) is a dominant driver of index misassignment when sequencing barcode libraries and could explain why misassignment rates are higher for amplicon sequencing than sequencing more diverse libraries, e.g., RNASeq or whole genomes. This makes sense given template switching relies on shared regions of homology between swapped reads, which are prevalent in amplicon sequencing (Box 2). This finding reinforces the recommended best practice of using unique dual indexing (or nested unique dual indexing) for barcode libraries, even in cases where you are confident there are few unincorporated primers remaining in the library, as these cause index hopping (Fig. 3D**;** left) but not template switching (Fig. 3D; right).

### Motivation and Design for Studying Noise Introduced by Environmental Differences

Although we have shown that index misassignment on patterned flow cells can be a major source of noise, this did not cause the variation in fitness observed in Fig. 1. All experiments comprising Fig. 1 were performed either on an unpatterned flow cell or using the nested unique dual-index approach outlined above (Fig. 3A; right panel). Therefore, we continued investigating additional sources of noise that might cause the observed variation in fitness in Fig. 1.

There is evidence that the fitness variation in Fig. 1 might be caused by subtle environmental differences across replicates and batches. First, we know that there were slight differences in the setup of some of the 9 “batches” of experiments delineated by vertical lines (Fig. 1). For example, the set of replicates from 5/9/15 was uniquely performed without including an ancestral yeast strain, and the set on 8/17/15 was performed without one of the growth steps that precede the fitness measurement. Second, even when there are no documented differences in the experimental setup, there are bound to be minor differences that might affect how these yeast strains grow, especially considering that the experiments were performed by 4 different researchers, in 2 different buildings, and over a time span of 4 years. Third, the differences in fitness across replicates and batches do not appear to represent random noise as would be expected if they were caused by the stochastic sampling that happens independently for every replicate. Instead, *IRA1* and *GPB2* lineages go up or down in fitness together across replicates such that the difference in their fitness is more consistent across replicates (Fig. 1; std dev across purple diamonds is 0.032, while std. dev. across boxplot medians is 0.117 for *IRA1* and 0.09 for *GPB2*). This may imply that both types of mutant respond the same way to whatever environmental difference is most salient across these replicates. Additional evidence that the fitness variation across these 9 batches represents a response to environmental differences comes from our previous work (Kinsler et al. 2020). Here, we were able to predict fitness variation across these batches by modeling how mutant fitness changes across more intentional environmental perturbations. This provides support for the idea that batch effects represent subtle genotype-by-environment interactions, rather than random technical noise.

Despite how obvious it may be that some of the variation in fitness reported in Fig. 1 reflects environmental differences between these 28 experiments, we were surprised that the differences across replicates and batches are substantial enough that they are visible by eye. For example, in the batch of fitness competitions performed on 12/10/17, the average fitness advantage of *IRA1* mutations is significantly smaller than in the batch of competitions performed on 5/9/15 (Fig. 1; compare panels 1 v. 2). And in the 3 replicate fitness competitions performed on 5/1/15, the average advantage of *IRA1* nonsense mutations differs, with this advantage being significantly lower in replicate ‘C’ (Fig. 1; compare replicates within panel 3). Having often made meticulous efforts to reproduce important aspects of the experimental conditions across replicates and batches, we thought other sources of variation would be more prominent than so-called “batch effects.” A minor comfort is that others have struggled with the same difficulties (Lithgow et al. 2017; Worthan et al. 2023). A second reason we were surprised these batch effects were so salient is that they are so often overlooked when researchers average across replicates and batches to get more accurate fitness estimates. Doing so may inflate estimates of precision, and can even lead to erroneous conclusions (Box 1).

**Fig. 4:**
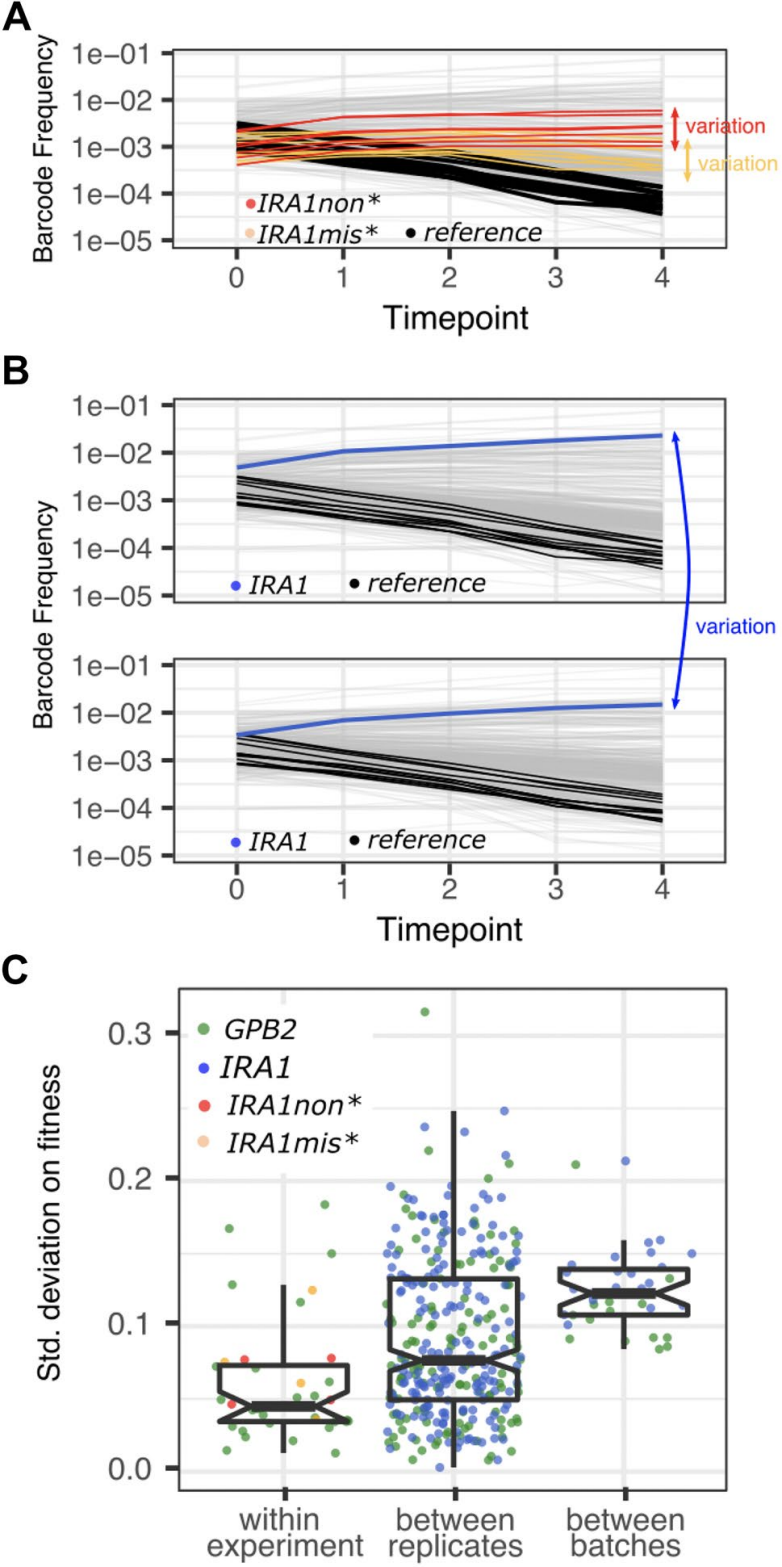
How fitness is estimated and how these estimates vary. **A** This panel clarifies how we study variation within an experiment by comparing the fitness of identical genotypes competing within the same vessel. We studied 6 strains that each possess a frameshift AT to ATT insertion at bp 4090 in *IRA1* (red) as well as 8 strains that each possess a G to T mutation at bp 3776 in *IRA1* (orange). The standard deviation on the fitness across these 6 or 8 identical genotypes is shown in panel C. **B** This panel clarifies how we study variation in fitness between batches and replicate experiments. To do so, we calculate how much variation exists in fitness estimates for a single unique barcode when measured in different experiments. **C** Each point represents the standard deviation across multiple fitness measurements. Standard deviations were calculated as follows: We calculate the standard deviation on fitness within an experiment for all lineages carrying any mutation in *GPB2* (green), carrying an AT to ATT insertion at bp 4090 of IRA1 (red), or carrying a G to T mutation at bp 3776 of *IRA1* (orange). We do this for each of 28 replicate experiments in which multiple *GPB2* lineages are present, and 4 experiments in which multiple IRA1non* or IRA1mis* lineages are present (for a total of 28 + 4 + 4 = 36 observations in this category). We calculate the standard deviation on fitness across replicate experiments for each of 21 barcoded lineages possessing mutations to *IRA1 and* 13 barcoded lineages with mutations to *GPB2.* We did this 9 times each (since there are 9 batches of replicates) for a total of 21 × 9 + 13 × 9 = 306 observations in this category. We calculate the standard deviation on fitness across batches by grouping measurements from all 28 replicate experiments whether or not they were performed in the same batch. The fitness of 21 barcoded lineages possessing mutations to *IRA1* was calculated, as was the fitness of 13 barcoded lineages with mutations to *GPB2* (34 observations in this category). For each category listed on the horizontal axis, boxplots summarize the same features as Fig. 1, with notches representing a roughly 95% confidence interval around the median calculated as 1.58 × IQR/√n

Given our surprise at the magnitude of these batch effects, we wanted to better understand how much of the variation in Fig. 1 is likely due to environmental differences between replicates and batches, as opposed to stochastic differences (sampling noise) that can be introduced at many stages of the experimental pipeline (e.g., when a subset of cells are sampled to start a liquid culture, when a subset of cells are sampled for DNA extraction, when a subset of barcodes are sampled for amplification via PCR, when a subset of barcodes are sampled for sequencing). Our strategy for disentangling these two types of noise is as follows. Variation in the fitness of identical mutants competing within the same vessel can be caused by all the aforementioned sources of sampling noise, but not by environmental differences. Thus, in order to understand how much additional noise environmental differences contribute, we compared variation in fitness of identical mutants competing within the same vessel (Fig. 4A) to variation in fitness of identical mutants across replicate competition experiments (Fig. 4B).

To study identical mutants competing within the same vessel, we utilized strains that were engineered to possess identical mutations in the *IRA1* gene, but were given different barcodes (Kinsler et al. 2020). We studied 6 such strains that each possess a frameshift AT to ATT insertion at bp 4090 in *IRA1* (referred to herein as ‘*IRA1*non*’) as well as 8 such strains that each possess a G to T mutation at bp 3776 in *IRA1* (called ‘*IRA1*mis*’) (Fig. 4A). We also utilized strains with adaptive mutations in *GPB2*, because previous work found that different mutations to this gene had similar effects on fitness, regardless of their position within the open reading frame (Levy et al. 2015; Venkataram et al. 2016).

### We Observe More Variation in Fitness Between Experiments than Within Experiments

Our fitness inference method yields different fitness estimates for identical mutants competing against one another in the same vessel (Fig. 4C; leftmost boxplot). For example, each red point in Fig. 4C represents the standard deviation in fitness across 6 barcoded yeast strains possessing identical frameshift mutations in the *IRA1* gene. Since these mutants were only included in each of the four replicate experiments performed on 12/10/17, there are 4 red points. Each green point in the leftmost boxplot of Fig. 4C represents the standard deviation in fitness across 13 barcoded yeast strains possessing different mutations in the *GPB2* gene. These lineages are included in all 28 replicates, so there are 28 points. While some of these 28 experiments seem to have suffered higher levels of sampling noise than others, on average the amount of noise observed across lineages competing within the same environment tends to be less than the amount of variation observed across lineages competing in different environments (Fig. 4C; notches indicate roughly 95% confidence intervals around the median). Across all replicate experiments and more so across replicates performed on different days (i.e., in different batches), we see additional variation in fitness beyond what we observed among identical mutants competing within the same vessel. In other words, batch effects are prevalent in our study. This observation of strong batch effects highlights how strongly the impact of mutations depends on context, including even subtle contextual changes that are present across replicates and batches (Eguchi et al. 2019; Geiler-Samerotte et al. 2020; Kinsler et al. 2020). It also reinforces the importance of a careful experimental design where the effects of treatment are not confounded with the effects of batch (Box 1).

The prevalence of batch effects in our study begs questions about to what extent they are present in other studies. One could argue that here we are comparing apples to oranges because there are known differences between some of our batches. For example, some experiments were performed without a pre-culturing step or have other differences (Fig. 1 & Table S1) (Kinsler et al. 2020). Perhaps, had the experiment been reproduced without deviation, the batch effects would be less salient. However, variation across experiments would not disappear. Even across identical replicate experiments performed on the same day, we see significantly more variation than observed within an experiment (Fig. 4C; compare leftmost and middle boxplots). This highlights that fitness is sensitive to subtle differences between putatively identical experiments that are difficult to control. Similarly, when we omit the 3 batches that involved the protocol modifications described in Fig. 1, batch effects are just as severe (median standard deviation remains at 0.12 as it is in the rightmost boxplot on Fig. 4C). These observations make it difficult to pinpoint what causes replicate and batch effects in our study. When we consider the laundry list of tiny ways an experiment might differ from one day to the next (see methods & Table S1), it seems like many of these sources of variation are present in other studies. Thus, our goal is to generate discussion on how to best proceed given our finding that fitness may be extremely sensitive to environmental and/or procedural differences between experiments that are difficult to avoid. Perhaps our finding inspires new best practices in experimental evolution that include better reporting (and adhering to) an invariant experimental pipeline. Or perhaps, we might embrace the idea that differences between experiments are bound to happen and think about what we might gain by explicitly studying how fitness changes across replicates and batches (see discussion).

One caveat is that, in addition to environmental differences between batches, another potential source of batch effects may be variation in the frequency of baseline reference strains used by the inference method to calculate the fitness of each mutant (Venkataram et al. 2016; Li et al. 2018a; Kinsler et al. 2020). One key step of the fitness inference method determines either the mean fitness of the population (Venkataram et al. 2016; Li et al. 2018a) or the fitness of spiked in reference strains (Kinsler et al. 2020). Then, it sets all other fitnesses by these initial baseline inferences (Box 3). If these initial inferences differ across replicates or batches, for example, due to stochastic sampling noise that affected the reference strains, it could lead to variation in inferred fitness values that preserve their rank order, as observed in Fig. 1 where *IRA1* mutants are always more fit than *GPB2*. Thus, another way to improve precision in fitness estimation may be to improve the accuracy of these initial inferences by spiking in a larger number of reference strains. However, differences in mean fitness inference are unlikely to entirely explain batch effects. If they did, we might expect to see the same amount of variation across replicates and batches. However, variation across batches tends to be greater (Fig. 4C). Also, previous work suggests that the batch effects we study here indeed reflect subtle genotype-by-environment interactions (Kinsler et al. 2020).

In sum, batch effects, and to a lesser degree, replicate effects, appear to be prevalent in barcoded fitness measurements (Fig. 4C). They are likely caused by environmental differences (e.g., subtle differences in the media, equipment, strain composition, and/or protocol). On one hand, batch effects may represent a latent opportunity to learn new biology, e.g., which mutants are most sensitive to which environmental perturbations. On the other hand, batch effects can be extremely problematic in some circumstances. For example, they can make typically reported measures of precision, such as percent error across replicates, difficult to interpret (Box 1). And they can unfairly exaggerate fitness differences between environments that were studied on different days or conversely, they can obscure these differences (Box 1). The severity of the problem depends on the magnitude of the fitness differences that the researcher is hoping to capture. In the field of experimental evolution, where researchers are often interested in understanding subtle fitness differences across environments (Venkataram et al. 2016; Li et al. 2018b, 2019; Jerison et al. 2020; Kinsler et al. 2020; Boyer et al. 2021; Bakerlee et al. 2021), batch effects may represent an important and previously underappreciated confounding factor.

## Discussion

Here, we analyzed sources of noise that contribute to variation in barcoded fitness measurements. We found that technical noise due to DNA extraction and PCR amplification had small contributions to measurement noise when they were performed via the methods we used (Fig. 2). And we found that another technical source of noise, template switching between sequencing reads from different samples, can provide a more major source of noise (Fig. 3). One of our most upsetting observations was that fitness varies so much across replicates and batches (Fig. 4). Taken at face value, the observation that the fitness of an organism can change so much between replicate experiments raises questions about our ability to obtain and interpret fitness measurements. This concern is relevant in many fields, including experimental evolution (Levy et al. 2015; Li et al. 2019; Boyer et al. 2021; Aggeli et al. 2021; Bakerlee et al. 2021), deep mutational scanning (Fowler and Fields 2014), and high-throughput genetic engineering systems (Sharon et al. 2018; Bakerlee et al. 2022).

One way to contend with this concern is by reducing comparisons across replicates and especially batches as much as possible. For example, the fitness of all mutations could be measured at the same time in the same flask to eliminate replicate and batch effects. In cases where this is not possible, for example, when comparing across two or more different environments, all experiments could be performed in the same batch, with as many factors held constant as possible (incubator, flask shape, base media, etc.). Indeed, this is how we performed the #1BigBatch experiment that inspired this manuscript. But performing all experiments in one big batch is difficult and further, does not address the issue of reproducibility across labs. Should scientists studying fitness consider investing in ultra-precise scales for use during media prep or incubators with tight temperature control to ensure that others can more accurately recreate their work? Should the methods sections of relevant articles be greatly expanded to include details like whether fresh media was made each time and warmed up before adding cells to it? We hope that the results reported here, particularly the large amount of variation in fitness observed across batches, inspires deeper discussion of this important reproducibility issue.

On the other hand, perhaps batch effects need not be reduced. If the variation in fitness across replicates and batches does not reflect imprecise measurements, and instead results from small environmental differences between experiments, perhaps this is a time to turn lemons into lemonade. Perhaps we should consider fitness as a parameter that inherently varies and can never be fully replicated even in the same experiment. If we embrace this context dependency and report this variation instead of merely averaging away this signal, we might begin to glean new insights about these sensitive systems.

The observation that fitness is sensitive to subtle environmental changes across batches contributes to a growing mountain of evidence that context dependency is pervasive in biological systems (Eguchi et al. 2019; Liu et al. 2020; Kinsler et al. 2020; Bakerlee et al. 2021). This rampant context dependency raises many philosophical issues about how to define an ‘environment.’ Should we think of each batch as a new environment or each replicate? When we perform an evolution experiment, do we expose our evolving population to a new environment every time we transfer them into new media? Is the environment also defined by which other genotypes are present and at what frequencies? Stepping outside of laboratory experiments, how should we think of the fitness of an organism if it is extremely sensitive to the small changes in the environment that happen over the course of the day? In particular, should we re-evaluate the use of models in which an organism has a single selection coefficient and perhaps define a range of coefficients that pertain to the relevant range of environments it experiences?

In sum, improvements in our ability to measure fitness have allowed us to detect rampant context dependency hiding in a high-replicate dataset. Ironically, high-replicate datasets are often generated to improve measurement precision rather than reveal the inherent imprecision. The revelation that fitness is inherently varied across replicate experiments inspires new goals for fitness measurements going forward. In addition to measuring fitness precisely by eliminating technical sources of noise, the community must think carefully about how to measure an extremely context-dependent parameter in a reproducible way, how to report precision on these measurements, and how to use these measurements to understand and model processes, such as natural selection, which can detect fitness differences orders of magnitude smaller than those we observe in the lab and thus may be even more sensitive to small environmental fluctuations.

## Boxes

**Fig. B1:**
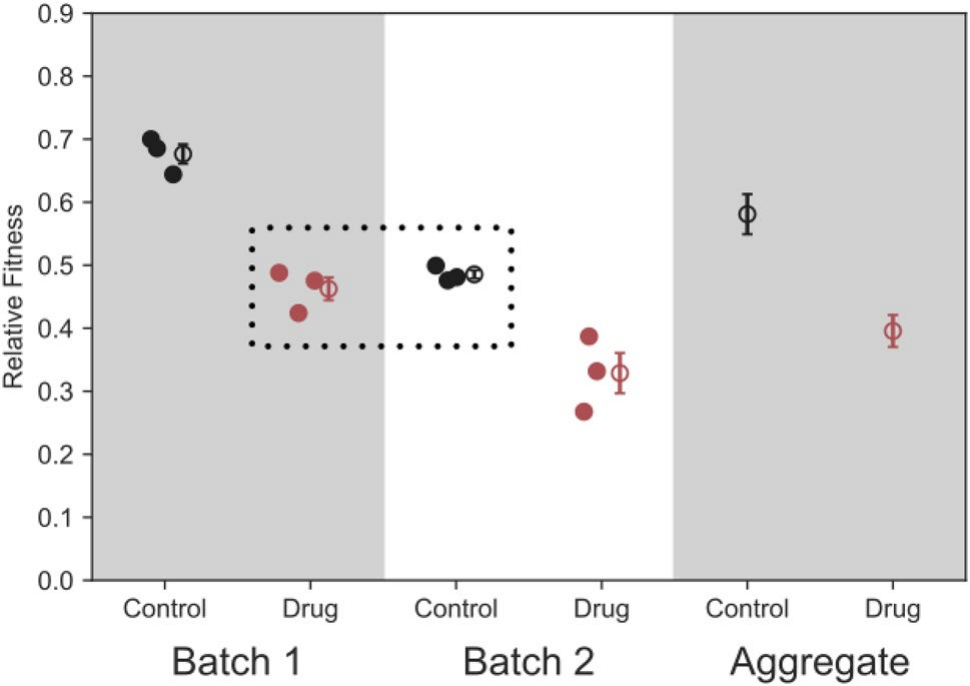
Problems introduced by batch effects. This figure shows a toy example using simulated data that reflects how aggregating across measurements from different batches (e.g., measurements performed on different days) can ignore batch-to-batch variation leading to an overestimate of measurement precision. Each of the 3 dots represents replicate fitness measurements of a single genotype. In this example, batch effects are at least as strong as the effect of the drug on fitness. Since both drug and control conditions were included in each batch, the aggregate panel accurately captures the fitness difference between the drug and control, but overestimates the precision of this measurement. Had the experiment been designed differently, with drug and control conditions being surveyed on different days (dotted box), the effect of the drug on fitness may have been obscured by batch effects

Box 1—Improving Precision by Adding more Replicates can Lead to Erroneous Conclusions Improving fitness measurement precision by adding more replicates makes several assumptions that may not be true, especially if replicates were performed on different days (i.e., in different “batches”). For example, this method assumes that between-replicate (or between-batch) variation is uncorrelated technical noise, rather than variation in fitness due to environmental differences. For example, consider a case where fitness has been measured in a control condition and in the presence of a drug (Fig. B1). If we fail to consider the effect of hidden variables that vary from batch to batch, then in this case we would correctly infer that the fitness is higher in the control condition when we take the average across all replicates and batches (Fig. B1; rightmost ‘aggregate’ panel). Our confidence in the precision of our fitness measurements (as measured by standard error—$\frac{\sigma }{\sqrt{n}}$), would be fairly high, given the relatively large number of replicates across all batches. However, if we had measured fitness in the drug condition only in batch one and the control condition only in batch two (Fig. B1; dotted box), we would have incorrectly observed that the drug has no effect on fitness. In this case as well, our confidence in this fitness measurement would be fairly high, given we performed 3 replicates for each condition. In both cases, our confidence in the measurement is overstated, given the standard error may be over-representing how precisely fitness can be measured, as there are uncontrolled factors that vary from batch to batch that change the fitness effects of the mutant across the environments (Fig. B1).

Box 2—The Consequences of Template Switching on Barcoded Competition Experiments Given that template switching seems to occur more in barcoded libraries than in RNASeq or WGS data, we suspect that it is facilitated by homology among reads. Because of how barcode regions are synthesized and cloned, there are often fairly long stretches of homology flanking the barcode region. Thus, one potential area of future study is to assess the extent to which the length of homology contributes to the prevalence of index misassignment due to template switching. Furthermore, future barcoding designs may want to consider reducing the amount of constant sequence both in the barcode construct itself and the primer sequences used to reduce the rates of template switching (Hegde et al. 2018; Johnson et al. 2023). Doing so is important not only to avoid wasting sequencing reads on swapped samples, but also to minimize swapping in cases where unique dual indexing cannot detect it.

**Fig. B2:**
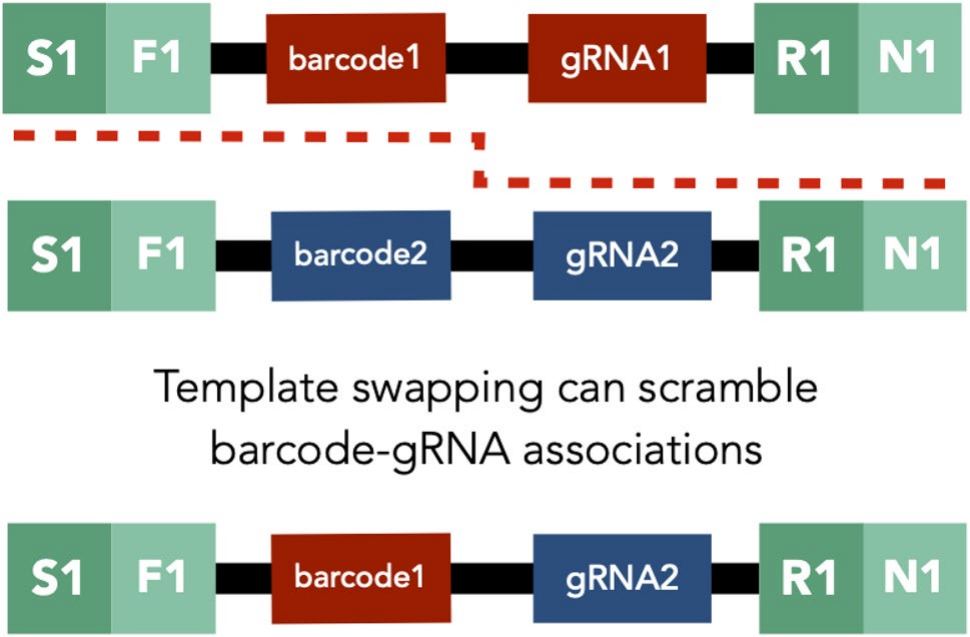
Template switching can scramble internal associations on different ends of the read. This figure shows a schematic of a template switching event occurring between two reads belonging to the same sample for an application where barcodes are associated with guide RNAs (i.e., barcoded CRISPR screen). In this case, template switching (denoted by the red dashed line) that occurs in the homologous region in the middle of the read would cause the gRNA-barcode associations to be scrambled. This would not be identified as a swapping event using unique dual indices because both reads belong to the same sample and have the same indices

While unique dual indexing allows removal of template switching events between timepoints or samples, it does not detect swapping events between within the same sample. These “hidden” swapping events result in chimeric reads and can cause issues for certain applications. For example, in some methods including massively parallel reporter assays, barcoded or combinatorial CRISPR screens, and double-barcode systems, it is important to associate information from one side of the read with the other (Fig. B2) (Wong et al. 2016; Adamson et al. 2016; Najm et al. 2017; Gordon et al. 2020). Researchers carrying out experiments that rely on these internal associations should utilize sequencing platforms with reduced template switching rates (e.g., Illumina non-patterned flow cell machines like NextSeq or MiSeq) and/or experiment with reducing homology among reads. Additionally, researchers may want to include explicit known barcode-gRNA pairs that allow for the detection of these “hidden” swapping events within a sample.

Box 3—Inference of a Fitness Benchmark is Critical to Reproducible Measurements During a fitness competition experiment, a strain’s change in frequency depends on its fitness relative to the fitness of the other strains in the population (Li et al. 2018a). Thus, benchmarking the fitness of a population is a crucial step to properly normalizing fitness measurements and ensuring reproducibility across environments and batches.

There are two common methods to calculate a fitness benchmark. One method directly calculates the mean fitness of an entire population using the inferred fitnesses and frequencies of strains in the population (Li et al. 2018a). This method is commonly used in screens where many mutations are being simultaneously assayed (Fowler and Fields 2014; Sharon et al. 2018; Liu et al. 2020), as it is assumed that most mutations have little to no effect on fitness such that this average is representative of the fitness of an unmutated strain. Another method for benchmarking fitness is the use of reference strains. With this approach, a reference strain/s is spiked into the population, and fitnesses of other strains are explicitly calculated relative to this reference. This approach allows fitnesses to be easily compared across environments, because they are always relative to the same reference strain. When studying the fitness of adaptive mutations, the reference strain is often the unmutated ancestor of an evolution experiment (Venkataram et al. 2016; Kinsler et al. 2020). Because this normalization affects the fitness of all other measurements, it is important that the ancestor’s change in frequency is measured well, otherwise it can serve as an additional source of noise and batch effects across experiments. Measuring the ancestor’s fitness can be difficult because this ancestor will often receive lower sequencing coverage as it falls in frequency while the adaptive mutants rise in frequency. Thus, it is advisable to spike in the ancestor at a high concentration to ensure adequate coverage (and low sampling noise) and to include many uniquely barcoded copies to contend with variation in fitness arising from technical sources. Because of the potential advantages and disadvantages of both methods of benchmarking fitness, the field could benefit from future theoretical and experimental work to understand which methods are preferred for different types of experiments.

## Methods

### Fitness Measurements

The 28 replicate fitness measurements presented in Fig. 1 were described in a previous study (Kinsler et al. 2020). Briefly, growth competitions were setup between a pool of barcoded mutants and a reference strain. These 28 competitions were performed in 9 batches, which each were performed on a different date in one of two locations. Table S1 contains a list of the known differences between batches, including the number of barcoded yeast strains competing in each pool. But in any type of study, there are countless things that may differ between putatively identical experiments. A partial list of things that may (or may not) differ between fitness competition experiments includes the incubator used to keep cultures warm, the scale used to measure media components, the number of cycles used during PCR amplification, the number of barcoded strains in the competition, the number of reference strains used, the person performing the experiment, how fast that person transferred cultures from one time point to the next, the lab in which the experiment was performed, the humidity and temperature that day, whether or not fresh media was warmed or taken straight from the refrigerator when initiating a culture, the length of the preculture, and the number of timepoints for which barcode frequencies were tracked. Some of these differences may arise unintentionally due to stylistic differences between researchers and others can arise intentionally as researchers hone and improve their protocols.

These fitness competition experiments were monitored by sequencing the entire populations’ barcodes at 3 or more timepoints. The change in the frequency of each barcode over time reflects the fitness of the adaptive mutant possessing that barcode relative to the reference strain. After a growth competition is complete, DNA was extracted from frozen samples and resuspended in Elution Buffer to a final concentration of 50 ng/μL for later use in PCR reactions. A two-step PCR was used to amplify the barcodes from the DNA. The first PCR cycle used primers with inline indices to label samples. Attaching unique indices to samples pertaining to different conditions or timepoints allows us to multiplex these samples on the same sequencing lane. Each primer also contained a Unique Molecular Identifier (UMI) which is used to determine if identical barcode sequences each represent yeast cells that were present at the time the sample was frozen or a PCR amplification of the barcode from a single cell. The second step of PCR used standard Nextera XT Index v2 primers (Illumina #FC-131-2004) to further label samples representing different conditions and timepoints with unique identifiers that allow for multiplexing on the same sequencing lane. Samples were uniquely dual-indexed following the nested scheme in Fig. 3. Pooled samples were then sent to either Novogene (https://en.novogene.com/) or Admera Health (https://www.admerahealth.com/) for quality control (qPCR and either Bioanalyzer or TapeStation) and sequencing. Data were processed by first using the index tags to de-multiplex reads representing different conditions and timepoints. Then, reads were mapped to a known list of barcodes, PCR duplicates were removed using the UMIs, and the frequency of each barcode was measured at each time point. Fitness was inferred from changes in barcode frequency over time using a modified version of fitness assay python (https://github.com/barcoding-bfa/fitness-assay-python) available at https://github.com/grantkinsler/BarcodersGuide, along with the rest of the code used in this study.

### Technical Replicates

To create technical replicates of the DNA extraction procedure, frozen cell samples from a given timepoint of a pooled fitness competition were divided in half prior to DNA extraction. The halved samples were then treated separately for all downstream steps. All sample preparation was conducted as described above and as detailed in Kinsler et al 2020, except in a few cases. In some of these exceptional cases, marked by a triangle in Fig. 2C, we omitted glass beads from one sample in the pair of technical replicates. In other exceptional cases, marked by a diamond in Fig. 2C, we added phenol to one sample in the pair of technical replicates. These modifications did not seem to affect reproducibility between the pair of samples (Fig. 2C). Similarly, for technical replicates of the PCR procedure, we divided samples in half after DNA was extracted and diluted to 50 ng/μL. These samples were processed independently for all downstream steps, following identical procedures as described in Kinsler et al 2020 with a few exceptions. In some samples, marked by a circle in Fig. 2C, we reduced the cycle time for one sample in the pair of technical replicates from 27 to 23 cycles. This did not affect reproducibility between a pair of samples (Fig. 2C). All technical replicates were performed on samples from the batch of pooled fitness competitions initiated on 12/10/17; this was the batch of experiments that we live tweeted about at #1BigBatch.

### Quantifying the Effects of Index Misassignment

Below, we describe the methods we used to index samples, identify index misassignments, and understand the mechanisms underlying index misassignment. Note that other than the section *Template switching on patterned flow cells appears to be a major source of noise*, results presented throughout the rest of the study were sequenced to minimize the amount of index misassignment as possible. In particular, we included only samples that were sequenced on non-patterned flow cell technology (HiSeq 2000, HiSeq 2500, or NextSeq) or were sequenced on patterned flow cell technology (patterned flow cell HiSeq X) with nested unique dual indexing.

We performed nested unique dual indexing following the method we developed in previous work (Kinsler et al. 2020). Briefly, this approach uses a combination of inline indices attached during the first step of PCR (12 forward and 8 reverse), as well as Illumina indices (12 Nextera i7 and 8 Nextera i5) attached during the second step of PCR. The latter indices are not part of the sequencing read (they are read in a separate Index Read). To uniquely label each sample, we use combinations of the Illumina and inline primers such that each of the 12 forward inline (F) indices is used solely with one Nextera i7 (N) index and each of the 8 reverse inline (R) indices is used solely with one Nextera i5 (S) index. We can then combinatorially utilize these 12 F/N combinations with the 8 R/S combinations to uniquely label up to 96 samples (Fig. 3).

To estimate the magnitude of index misassignment for a large set of samples across different sequencing platforms, we first constructed a library by pooling 95 samples that were uniquely dual indexed in this manner. We then sequenced this same library on two lanes, one a NextSeq lane and the other a HiSeq X lane. During the processing of the sequencing data, we mapped reads to all possible combinations of indices and counted the number of mapped reads to each index combination. Any reads which mapped to incorrect index combinations were then classified as misassigned.

To demonstrate an example of the effect that unidentified index misassignment can have on frequency trajectories and the calculation fitness, we used samples from replicate D of the experiment conducted on 12/10/17. The results depicting nested dual indexing use the reads to these samples from the HiSeq X run. To show what would happen if only combinatorial indexing were used, we ignored the inline indices and added up all the mapped reads to each barcode that mapped to the proper Illumina indices (N and S) alone. We then calculated fitness using these new reads.

To quantify whether index misassignment occurs most frequently via template switching or single swaps, we separately pooled a set of 8 samples, each containing their own unique index at all positions (except on particular S index—see note below) and sequenced this on a lane of HiSeq X. We then mapped these reads to every possible index combination. Reads were classified as a template switching event if the indices on both sides of the molecule matched proper samples but should not exist together (Fig. 3D). Reads were classified as a single swap if all indices matched a true sample except one. If the incorrect index was an Illumina index (N or S), it was classified as a single Ilumina swap, otherwise, if the incorrect index was in the read, it was classified as an inline single swap. Finally, if two indices were incorrect but the index combination was not due to a template switching event in the middle of the molecule, it was classified as a double swap.

Note that during the library creation for these samples, S513 was incorrectly used instead of S517 for four samples and S517 was used instead of S513 for four samples. For analysis about the general rate of index misassignment for amplicons, this may slightly decrease our estimated rate of index hopping, as single swaps between S513 and S517 would not be detected for the samples where the index was incorrectly used (likewise, single inline swaps between the corresponding inline R301 and R304 indices that are supposed to be uniquely associated with these samples). Note that this only applies to a small number of the total combinations that can arise via index misassignment, so the impact of this error should be minor. Only one of these samples was included in the library used to identify the mechanisms underlying index misassignment for amplicons. This resulted in the S517 index represented twice in this sample rather than only once. This could result in a slight under-estimation of the number of single swap events with Illumina primers (as again a single swap between the S517 index of these two samples would not be distinguished). Additionally, this error results in two primer combinations which could be either the result of single inline swaps or template switching. As template switching events are over 10 × more likely than single inline swaps according to our data, we assigned these ambiguous combinations as template switching events. Because these events are much more common and this only applies to 2 of the 56 possible combinations that result from template switching, this should result in only a minor misestimation of the template switching and inline single swap rates.

## Supplementary Information

Below is the link to the electronic supplementary material.

Supplementary file1 (DOCX 12 kb)
