# Supplementary material for: Extreme Sensitivity of Fitness to Environmental Conditions: Lessons from #1BigBatch
Source: J Mol Evol. 2023 May 27;91(3):293–310. doi: 10.1007/s00239-023-10114-3 (PMC10276131; doi:10.1007/s00239-023-10114-3)
Supplement: Supplementary file 1 — Supplementary file1 (DOCX 12 kb) [file 239_2023_10114_MOESM1_ESM.docx]

| **Experiment Date** | **Description of known manipulation** | **Number of barcoded clones** | **Number of replicates** | **Experiment ID** |
| --- | --- | --- | --- | --- |
| 12/10/17 | Inclusion of IRA1 reference lineages | 500 | 4 | BB |
| 5/9/15 | No barcodeless ancestor | 4800 | 3 | 19 |
| 5/1/15 |  | 4800 | 3 | 13 |
| 8/17/15 |  | 500 | 3 | 21 |
| 5/9/15 |  | 4800 | 3 | 18 |
| 8/17/15 | No preculture | 500 | 3 | 20 |
| 9/4/15 | No preculture | 500 | 3 | 23 |
| 12/26/14 |  | 4800 | 3 | 6 |
| 11/24/14 |  | 4800 | 3 | 3 |

**Table S1.** A list of all experiments included in this study. We list known differences between the experiments, such as number of barcoded clones, pre-culture status, inclusion of reference lineages, and the ancestor.
